# Update of Key Clinical, Histological and Molecular Features of Malignant Bone Tumours Arising in the Craniofacial Skeleton

**DOI:** 10.3389/fonc.2022.954717

**Published:** 2022-07-07

**Authors:** Simon Haefliger, Vanghelita Andrei, Daniel Baumhoer

**Affiliations:** Bone Tumour Reference Centre, Institute of Medical Genetics and Pathology, University Hospital Basel, University of Basel, Basel, Switzerland

**Keywords:** genetics, craniofacial skeleton, malignant bone tumour, sarcoma, diagnostics

## Abstract

The craniofacial skeleton is a highly complex and specialized anatomic region containing and protecting the brain and sensory organs. Bone sarcomas arising here comprise a heterogeneous group of tumours, some of which differ in their biological behaviour compared to their peripheral counterparts. The reasons for this seem to lie, at least partially, in the embryonal development of the craniofacial bones. For reaching the correct diagnosis as the cornerstone of optimal personalised treatment planning, a multidisciplinary team of specialists, including pathologists, radiologists, oncologists, and head and neck surgeons needs to be involved. The most common tumours arising in the craniofacial bones are bone-forming tumours, cartilage-forming tumours, fibro-osseous lesions, giant cell-rich lesions, and notochordal tumours. While morphology remains the backbone for the diagnosis, the last decade has witnessed tremendous advances in the molecular characterization of tumours, and molecular testing is increasingly becoming a part of the diagnostic process. The integration of these new molecular markers into the diagnostic approach has undoubtedly increased the diagnostic accuracy and objectivity, and holds great promise to also identify new therapeutic targets for precision medicine in the future. Examples include *HEY1-NCOA2* in mesenchymal chondrosarcoma, *IDH1/2* mutations in chondrosarcoma and *TFCP2* rearrangements in rhabdomyosarcoma. In this article, key clinical, histological and molecular features of malignant bone tumours arising in the craniofacial region are discussed, with a special focus on the differential diagnosis and prognostic considerations.

## Introduction

The craniofacial skeleton is a highly complex and specialised anatomical region and the diagnosis of malignant bone tumours arising from this region represents a challenge for pathologists as well as for radiologists. Interestingly, some sarcomas originating from the craniofacial bones have different clinical and morphological characteristics compared to their peripheral counterparts. An accurate diagnosis is crucial for optimal clinical decision-making and an interdisciplinary approach taking into account also the corresponding imaging and clinical data is mandatory for an adequate classification of head and neck sarcomas. In recent years, the identification of new molecular markers provided an additional diagnostic layer that can be helpful to classify and objectify these generally rare tumours.

In this review, we will discuss key clinical, histological and molecular features of malignant bone tumours arising in the craniofacial skeleton. The following entities will be covered: conventional chondrosarcoma, mesenchymal chondrosarcoma, chordoma, osteosarcoma (high- and low-grade), *TFCP2* rearranged rhabdomyosarcoma, odontogenic sarcoma, and odontogenic carcinosarcoma.

## Chondrosarcoma

**Figure 1:**
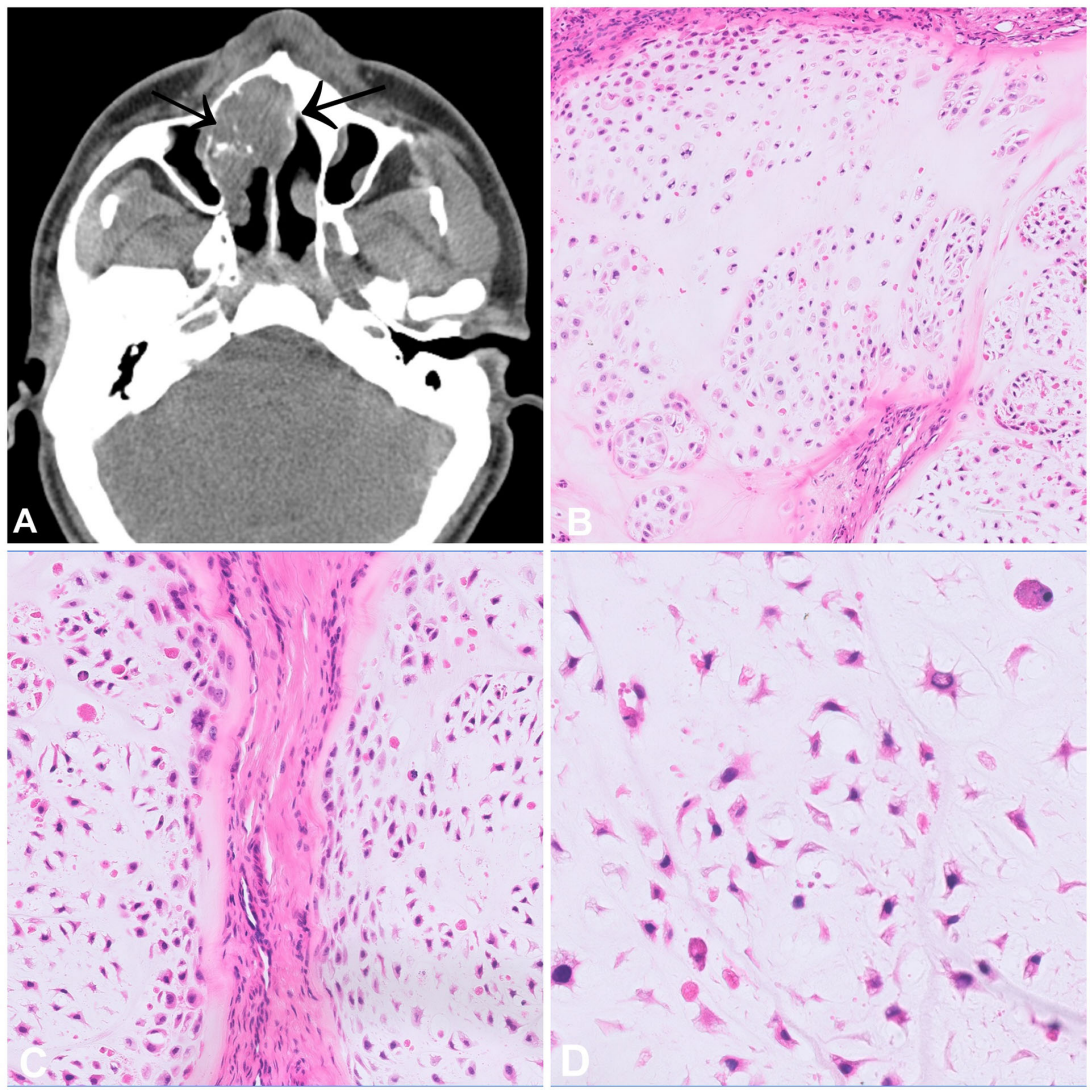
Chondrosarcoma. **(A)** CT scan of a high-grade chondrosarcoma arising from the nasal septum with focal mineralisation. **(B–D)** Grade 2 chondrosarcoma showing increase in cellularity when compared to a chondrosarcoma grade 1. The cells have atypical, hyperchromatic nuclei and are set in a hyaline matrix with myxoid change.

Chondrosarcoma is a malignant bone tumour which produces a cartilaginous matrix. In the head and neck, this tumour type is exceptionally rare, which may be influenced by the development of the craniofacial skeleton mainly through intramembranous ossification. Craniofacial chondrosarcoma occurs across all age groups, and the most frequently affected bones are the maxilla, the nasal septum and the skull base (1, 2). From a radiological point of view, these tumours range from well-defined lesions with popcorn-like matrix mineralization to large, destructive, and ill-defined masses (**Figure 1A**). Histologically, chondrosarcomas, as in other anatomical sites, consist of lobules of cartilage with entrapped host bone and/or bone permeation. Chondrosarcoma grade I resembles normal hyaline cartilage with only slightly increased cellularity and tumour cells showing small and uniform nuclei with dense chromatin, whereas chondrosarcoma grade II and III are characterized by increasing nuclear atypia, prominent nucleoli and less obvious chondromatous differentiation. Mitotic figures can also be found, the cellularity is generally more dense (**Figures 1B–D**). It should be noted that distinguishing between grade I and II chondrosarcoma can be challenging. The main differential diagnosis to consider is chondroblastic osteosarcoma, which is much more common in the craniofacial bones than chondrosarcoma. A meticulous search for even small amounts of neoplastic osteoid formation by the tumour cells as the defining feature of osteosarcoma is thus mandatory to establish the diagnosis of chondrosarcoma. In addition, approximately half of chondrosarcoma cases carry an *IDH1* or *IDH2* mutation, and therefore mutational testing can be helpful to verify the diagnosis and to exclude chondroblastic osteosarcoma (3). Isocitrate dehydrogenase (IDH) is a key enzyme in the tricarboxylic acid (= Krebs) cycle which converts isocitrate to alpha-ketoglutarate. In the mutant state, this enzyme acquires the ability to convert α-ketoglutarate into D-2-hydroxyglutarate (D-2-HG), an oncometabolite which impacts lipid synthesis, glycolysis and glutamine metabolism (4, 5). Interestingly, depending on the location, the mutational status of IDH seems to differ in craniofacial chondrosarcomas. Indeed, in the most comprehensive study to date, skull-based chondrosarcomas showed a high mutation rate (85.7%) while a small cohort of septum-based and maxillary chondrosarcomas (n=9) failed to show any mutations (6). These differences may be associated with the type of ossification involved in the development of the underlying bones (endochondral versus endomembranous). The main prognostic factors of chondrosarcomas of the craniofacial bones are histological grade and completeness of resection (1, 2). Dedifferentiation in the head and neck region is rare.

## Mesenchymal Chondrosarcoma

**Figure 2:**
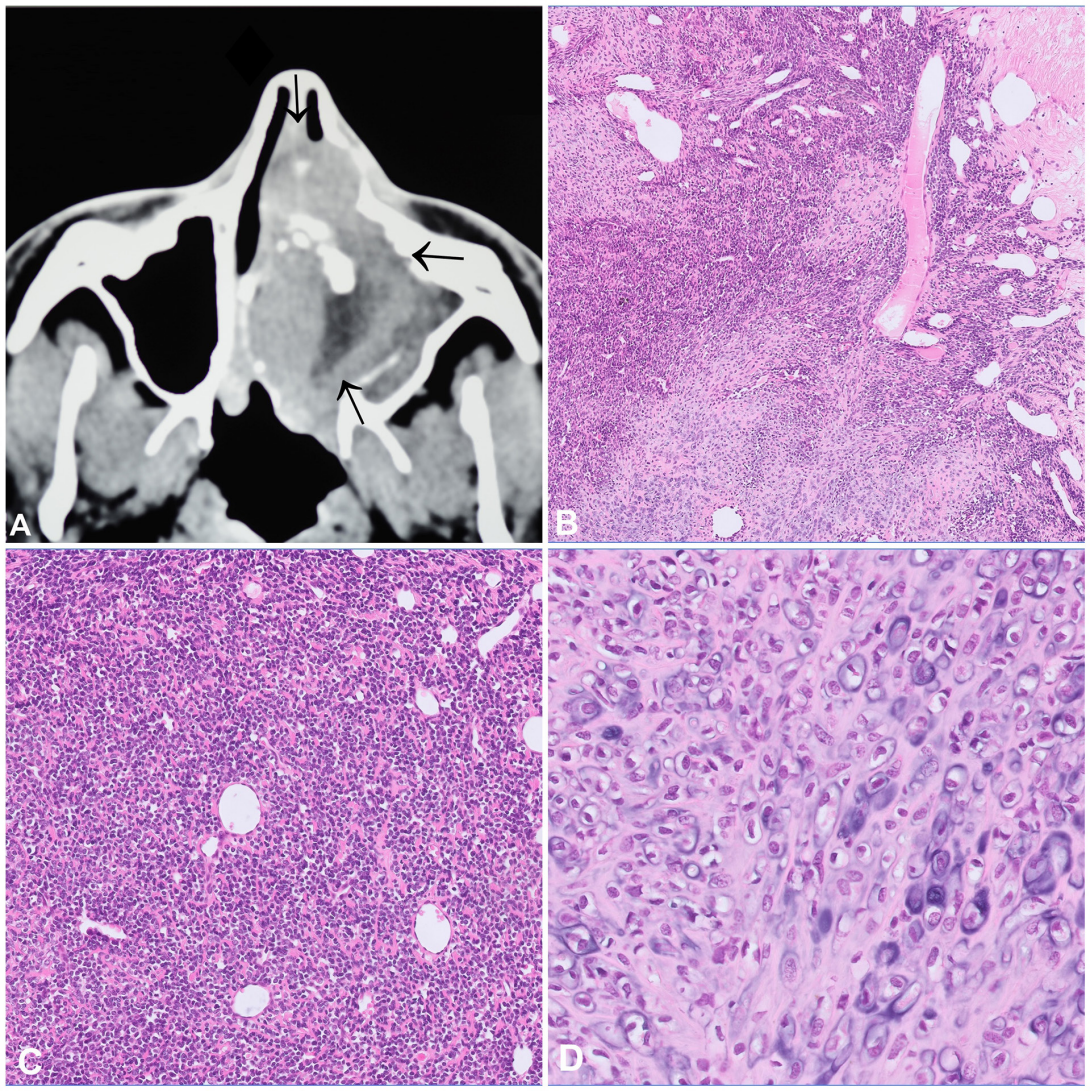
Mesenchymal chondrosarcoma. **(A)** CT scan showing an aggressive tumour of the nasal cavity with internal focal mineralisation, osteodestruction, and infiltration into the adjacent structures. **(B–D)** Mesenchymal chondrosarcoma with a characteristic biphasic pattern consisting of an undifferentiated small cell component with a characteristic haemangiopricytoma-like vascular pattern **(B, C)**, merging with islands of mature appearing cartilaginous component **(D)**.

Mesenchymal chondrosarcoma (MCS) is a rare high-grade tumour that can occur within bone (60%) and in the soft tissues (40%). Intraosseous MCS most commonly affect the craniofacial bones, particularly the jaws, and patients are mostly in the third decade of life (7, 8). Radiologically, these tumours present as large, osteolytic, and destructive masses with common extraosseous extension (**Figure 2A**). From a histological point of view, MCS has a prototypic biphasic pattern showing a combination of small round cells with haemangiopericytoma-like vessels admixed with lobules of mostly mature appearing cartilage which can exhibit calcification and secondary ossification (**Figures 2B–D**). The small round cells are similar to those of Ewing sarcoma. Immunohistochemistry (IHC) shows positivity for CD99 (membranous) and SOX9 (nuclear), only recently NKX3-1 has been suggested as a promising and highly specific marker for MCS (9). Virtually all MCS harbour a *HEY1-NCOA2* fusion gene, and only one single case originating in the soft tissue was reported to carry a *IRF2BP2-CDX1* (10, 11). The identification of these fusion genes can confirm the diagnosis, particularly in core needle biopsies lacking the differentiated cartilaginous component. The small round cell areas have been reported to show loss of TP53 expression, alterations of the RB1/E2F pathway and biallelic loss of *CDKN2A* (p16) (12). The differential diagnosis of MCS is broad and includes the whole spectrum of undifferentiated small round cell tumours, amongst other Ewing sarcoma, small cell osteosarcoma, and embryonal rhabdomyosarcoma. In addition, non-mesenchymal neoplasms like small cell carcinoma and lymphoma should also be considered in the differential. MCS is characterized by a high rate of local recurrence and metastasis, even after long disease-free intervals. The main prognostic factors are age, surgical resectability and anatomic site, with jaw tumours having the best prognosis (7). Neoadjuvant treatment regimens are generally recommended and targeted approaches might be a promising alternative in selected cases (13). We recently documented an impressive and sustained clinical response of a patient with widespread metastatic MCS following treatment with the multi tyrosine kinase inhibitor cabozantinib (manuscript in preparation).

## Chordoma

Chordoma is a malignant tumour that arises along the spine and recapitulates notochordal differentiation. The peak incidence is between the fifth and seventh decade of life. About a third of cases arise in the base of the skull, especially in younger patients, with the remaining lesions developing in the mobile spine and the sacrum (14, 15). According to the WHO classification, three subtypes can be distinguished: conventional, dedifferentiated and poorly differentiated, of which the poorly differentiated subtype has a predilection for young patients and a skull-based location (14). Some conventional chordomas show prominent areas of chondroid differentiation (so-called chondroid chordomas), thus the differential diagnosis includes highly differentiated chondrosarcoma. The characteristic feature of chordoma is the expression of brachyury, a transcription factor involved in notochordal development, which is also expressed in the chondroid areas, helping the distinction between these two entities (16). Chordomas also consistently express cytokeratins and S100 on immunohistochemistry (16).

**Figure 3:**
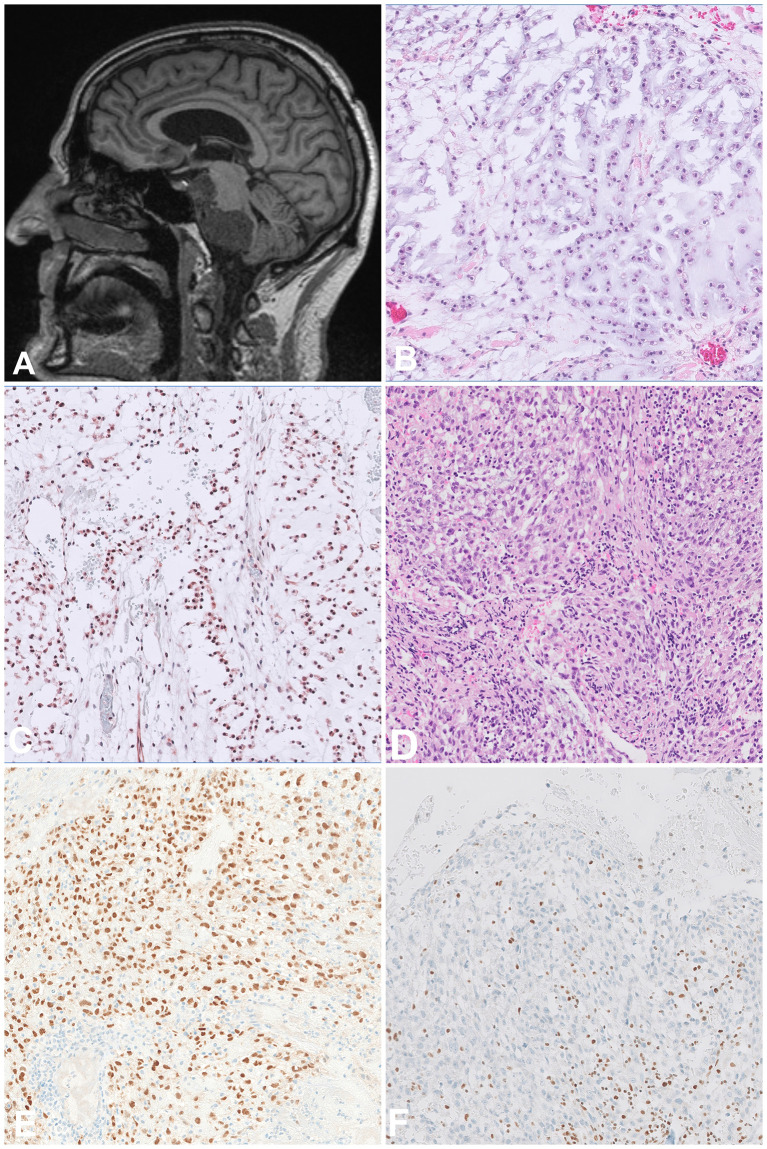
Chordoma. **(A)** Sagittal MRI scan showing a destructive tumour arising in the skull base and extending to the adjacent structures. **(B, C)** Conventional chordoma composed of atypical epithelioid cells arranged in chords and set in abundant myxoid matrix with a light basophilic appearance, the cells are positive for Brachyury. **(D, E)** Poorly differentiated chordoma composed of atypical epithelioid cells with a rhabdoid morphology and set in a more fibrous stroma, the cells are positive for Brachyury. **(F)** The tumour cells show lost expression of INI1, as opposed to background lymphocytes which can be used as positive control.

On imaging, chordomas are lytic, destructive masses and extension into the soft tissue is common (**Figure 3A**). From a histological point of view, conventional chordomas show lobules of epithelioid cells arranged in chords and nests with a multivacuolated cytoplasm (physaliphorous cells) and surrounded by an abundant myxoid matrix (**Figures 3B, C**). The dedifferentiated subtype has a biphasic appearance, showing the features of conventional chordoma adjacent to a high-grade sarcoma, mostly undifferentiated pleomorphic sarcoma (UPS) or osteosarcoma. Importantly, the dedifferentiated component does not necessarily express brachyury on immunohistochemistry. The classic physaliphorous cells and myxoid stroma are not usually seen in poorly differentiated chordomas, which tend to have a more rhabdoid morphology (**Figure 3D**) (14). In addition to the characteristic expression of brachyury (**Figure 3E**), poorly differentiated subtype show loss of *SMARCB1* (INI1) expression (**Figure 3F**). The differential diagnosis for this entity includes malignant rhabdoid tumour and rhabdoid meningioma. Due to their site of origin, chordomas are difficult to excise with wide margins, explaining the high rates of local recurrence. Conventional chordomas have a higher overall survival than both other subtypes (17). Also selected patients with chordoma might benefit from targeted treatment approaches as has been shown in individual case reports with CDK4/6 inhibitors (18).

## Osteosarcoma

**Figure 4:**
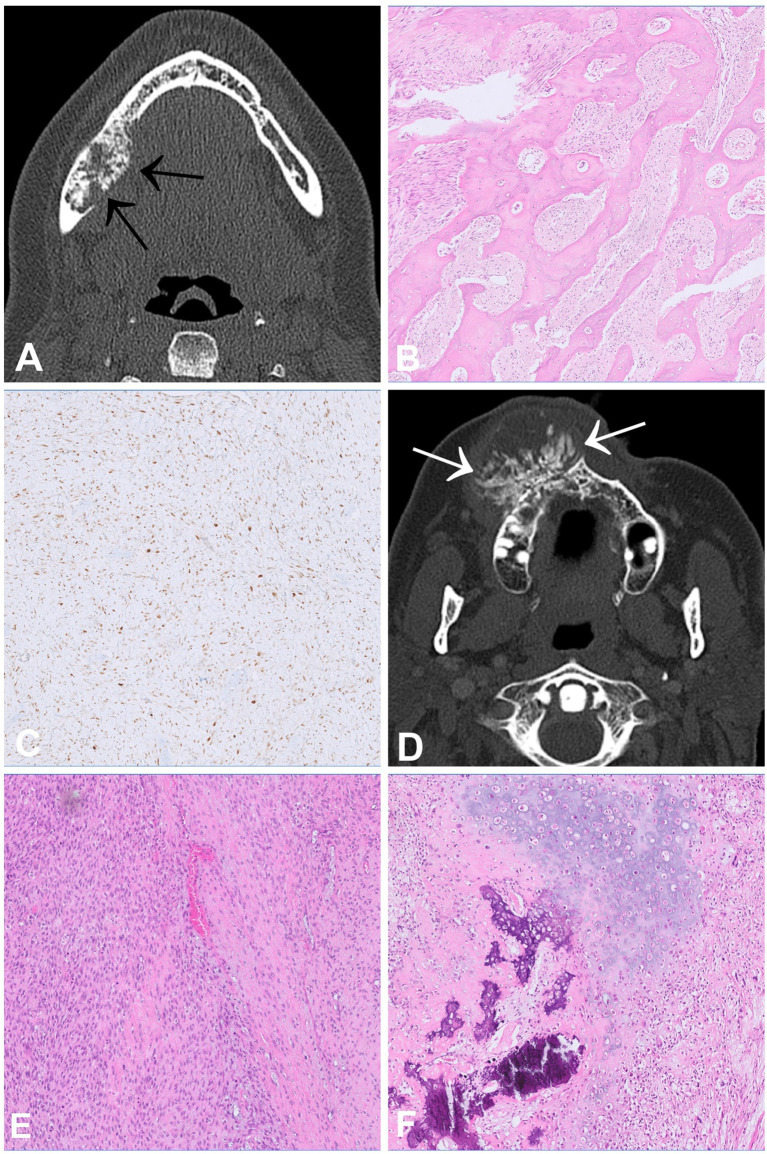
Low-grade central **(A–C)** and high-grade central **(D–F)** osteosarcoma. **(A)** CT scan showing a slightly expansive tumour within the right lower jaw with irregular mineralisation and aggressive growth, which permeates the cortex (arrows). **(B)** Histologically, low grade osteosarcoma shows irregular bone trabeculae embedded in fibroblastic stroma with only subtle atypia and low to moderate cellularity. **(C)** The tumour cells are posittive for MDM2 on immunohistochemistry. **(D)** CT scan showing a huge and aggressive mass with ossification arising in the right maxilla and bulging outward exophytically. **(E, F)** Morphological appearance of a high-grade osteosarcoma showing atypical spindle and epithelioid cells with associated malignant osteoid deposition and areas of cartilaginous differentiation **(F)**.

Osteosarcoma (OS) is the most common primary malignant tumour of bone and occurs most often in the metaphyses of long bones, mainly in children and adolescents. The jawbones, especially the mandible, represent the fourth most common site of OS following the distal femur as well as the proximal tibia and humerus. Compared to their counterparts in the peripheral skeleton, gnathic OS tend to occur one to two decades later in life and metastasize less frequently and later in the course of the disease (19). Radiologically, OS generally present as highly aggressive tumours with destructive growth, periosteal reaction and soft tissue extension (**Figures 4A, D**). CT is the modality of choice for staging and biopsy planning, whereas MRI is used to evaluate the precise intraosseous tumour extension and soft tissue involvement (20).

Conventional OS is a high-grade intra-osseous neoplasm composed of highly atypical cells producing neoplastic osteoid, which is the essential diagnostic feature. The tumours show osteo-destructive growth and entrapment of pre-existing bony trabeculae. Conventional OS can be subclassified into osteoblastic, chondroblastic and fibroblastic types according to their predominant type of matrix formation. The chondroblastic variant is most common in the jaws and can mimic chondrosarcoma, which is far less frequent in this location (**Figures 4E, F**). As mentioned previously, additional mutational testing for *IDH1* and *IDH2* can be helpful to differentiate between these tumours. Other OS subtypes include periosteal OS, which is of intermediate grade, as well as low-grade central and parosteal OS, which are both low-grade lesions. Periosteal OS demonstrate a predominant chondroblastic differentiation but are exceedingly rare in the craniofacial bones. Low-grade central and parosteal OS are generally characterized by immature trabecular bone formation embedded in fibroblast-like stroma with only subtle atypia and low to moderate cellularity. Whereas parosteal OS can show nodules or a cap of neoplastic cartilage, similar findings are rather unusual in central low-grade OS (**Figures 4B, C**). Distinguishing between low-grade OS and benign fibro-osseous lesions of the jaws, particularly craniofacial fibrous dysplasia, and sometimes cemento-ossifying fibroma, can be challenging, especially if the corresponding imaging is not available and/or the biopsy material is limited. We have recently described a new variant of ossifying fibroma occurring also outside of the jaws (ossifying fibroma of non-odontogenic origin) that can be another differential diagnosis to consider (21). However, osteosarcoma usually shows a more aggressive growth pattern on imaging and entrapment of host bone is never seen in benign fibro-osseous lesions, therefore representing a distinctive morphological feature. Chondromesenchymal hamartoma, usually occurring in the paranasal sinuses and in the nasal cavity can radiologically appear aggressive, and also show bone formation but affects a younger age group (most often <1 year, mean: 10 years). In addition, molecular testing can be useful to reach a reliable diagnosis. Amplification of *MDM2* which can be detected by fluorescence *in-situ* hybridization (FISH) is highly specific for osteosarcoma and absent in benign fibro-osseous lesions. The rate of MDM2 amplification in central low-grade OS varies between 30% in a larger study (5/17) and 100% in smaller case series. It is generally more common in parosteal OS (>85%). Both OS subtypes are exceptionally rare in the jaws (22–26). Positive staining with antibodies against *MDM2* and *CDK4* have also shown to be helpful in distinguishing low-grade osteosarcoma from benign fibro-osseous mimics but should be used with caution due to limited specificity (**Figure 4C**) (26). Additionally, mutational testing for *GNAS* can also be useful since *GNAS* mutations are specific for fibrous dysplasia and are not present in other fibro-osseous lesions, including low-grade osteosarcomas (24). In terms of pathogenesis, high-grade OS have, in contrast to low-grade OS, highly complex genomes with numerous structural and numerical aberrations (27). This complexity is most likely the result of single cataclysmic events such as chromothripsis or chromoplexy (28). The trigger driving these disruptive chromosomal alterations remains unknown but most likely involves inactivation of *TP53* (29). OS of jaws is associated with better prognosis compared to their peripheral counterparts, as they metastasize less frequently and later in the course of the disease (19). In contrast, extragnathic OS of the skull or facial bones behaves as aggressively as OS arising in the peripheral skeleton (30). Low-grade OS can usually be cured by complete resection without (neo-)adjuvant therapy, but long-term follow-up of the patients is recommended. For high-grade OS of the jaws, the benefit of chemotherapy is less clear than for tumours of the peripheral skeleton but might nevertheless improve the prognosis of patients and is generally recommended (31, 32). Histological grade and stage of disease are the main prognostic factors (30, 32).

## Rhabdomyosarcoma With *TFCP2* Rearrangement

**Figure 5:**
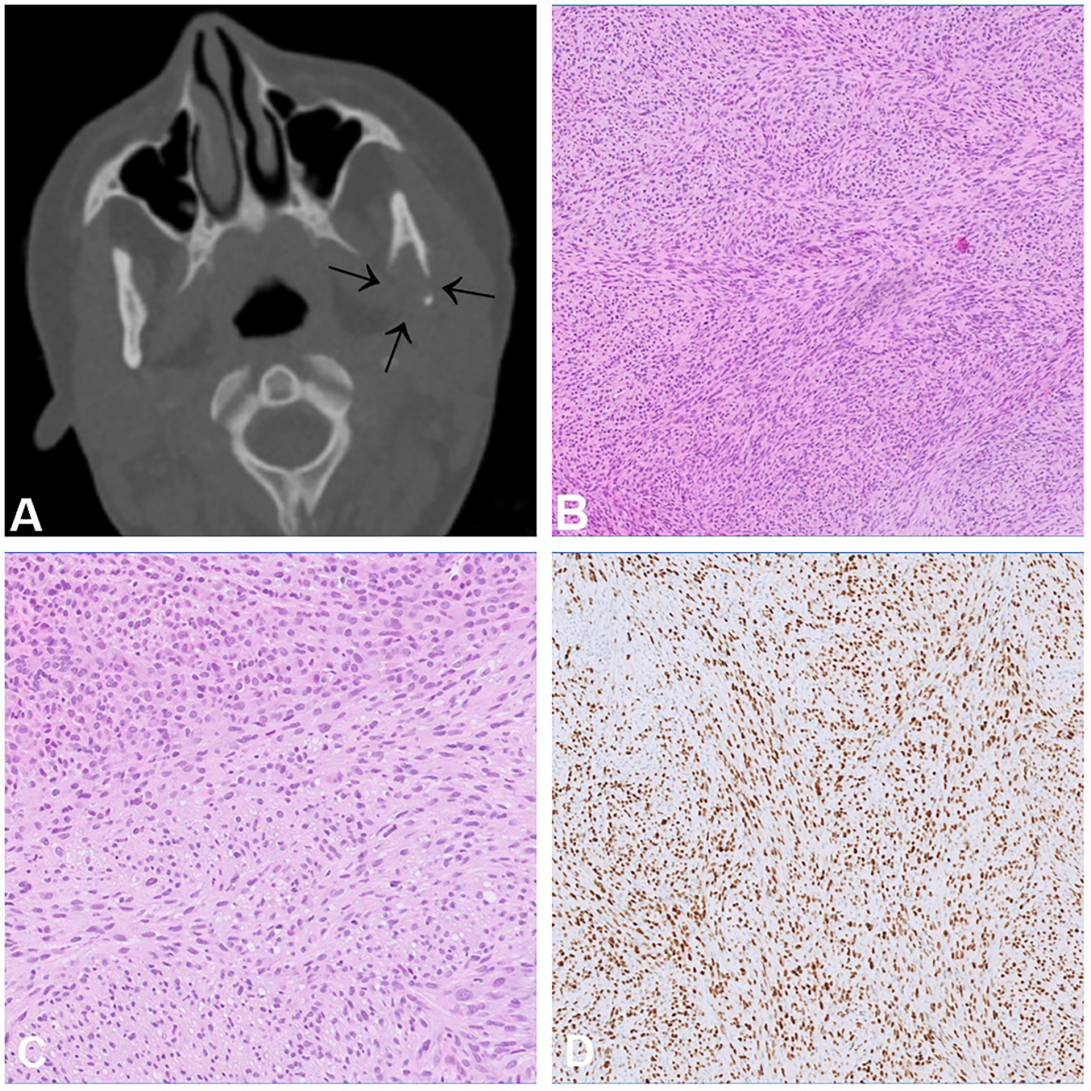
Rhabdomyosarcoma with TFCP2 rearrangement. **(A)** CT scan showing an aggressive and destructive tumour in the articular process of the left mandible. **(B, C)** Histologically, sclerosing rhabdomyosarcoma shows an admixture of spindle and epithelioid cells **(C)** arranged in solid fascicles with only scant stroma. **(D)** The tumour cells show diffuse positivity for MYOD1.

Rhabdomyosarcoma with *TFCP2* rearrangement (TFCP2-RMS) is a newly described bone sarcoma characterised by the fusion of *TFCP2* to either *EWSR1* or *FUS* showing a remarkable predilection for the craniofacial bones, mainly the mandible and the maxilla (33). They most commonly arise in bone and less frequently in soft tissue (34). The peak incidence is in the second decade of life (median age 25 years) (35). Radiologically, these tumours are large, poorly defined, and often extend into the surrounding soft tissues (34) (**Figure 5A**). Histologically, TFCP2-RMS is a biphasic neoplasm with a spindle cell and an epithelioid component arranged in solid sheets. In rare cases, only one of these components is present. The nuclei of the tumour cells are large, monotonous and show conspicuous nucleoli (**Figures 5B, C**). Necrosis and mitotic figures are common.The tumour cells are positive for cytokeratin (CK) AE1/AE3, desmin, myogenin, MYOD1 (**Figure 5D**) and ALK (33, 35). Due to the epithelioid morphology and positivity for CK, the differential diagnosis of TFCP2-RMS is broad and includes, amongst others, metastatic carcinoma, alveolar RMS and mesenchymal chondrosarcoma. The defining genetic feature of these tumours is the *TFCP2*-related fusion gene (33, 36) but apart from that they show complex genomic profiles. Homozygous deletions of *CDKN2A* are present in virtually all cases (33). Other genetic alterations include *MDM2* amplification, *TP53* mutations and upregulation of *ALK* expression (34, 35). The prognosis is poor, most of the patients present with locally advanced disease and distant metastases develop in more than half of the cases, either at the time of diagnosis or later in the course of the disease (34). The recurrence rate after surgery is high, even following (neo-)adjuvant therapy (37).

## Odontogenic Sarcoma and Carcinosarcoma

**Figure 6:**
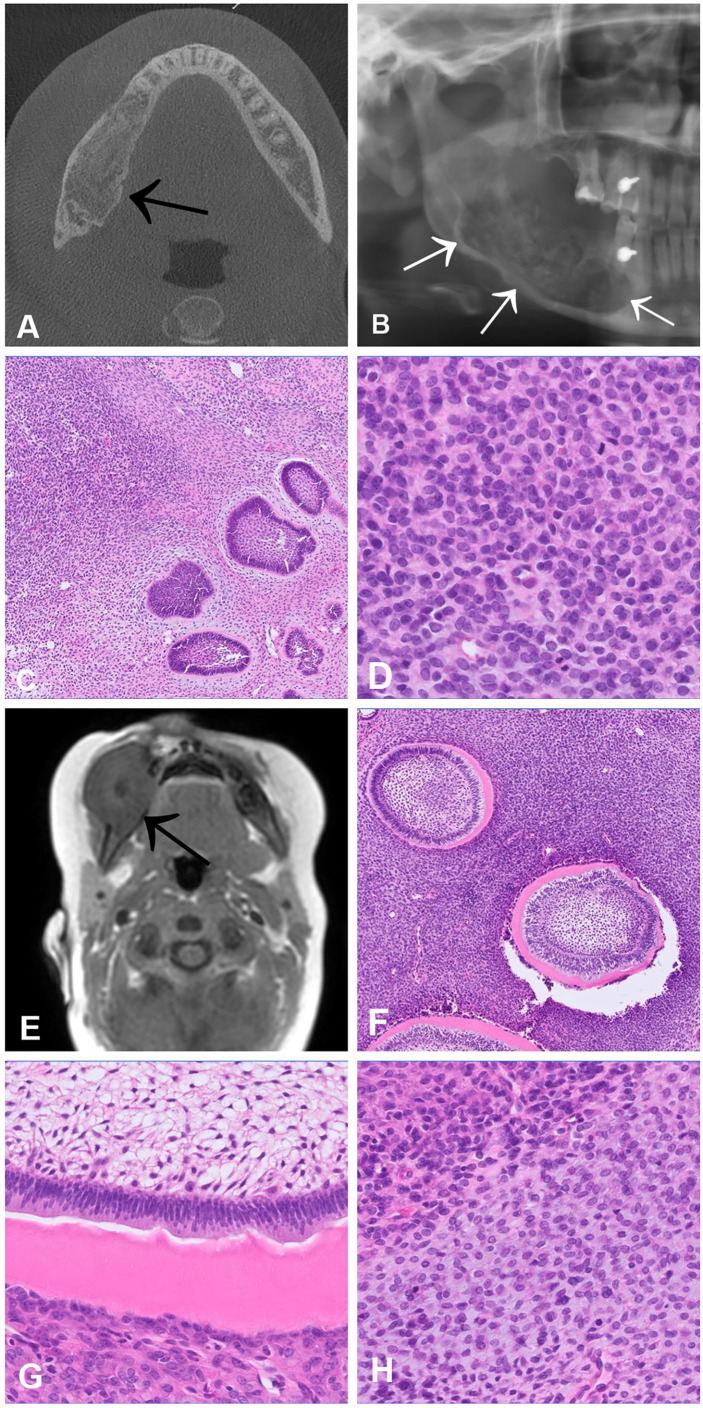
Ameloblastic fibrosarcoma **(A–D)** and Ameloblastic fibro-dentinosarcoma **(E–H)**. CT scan **(A)** and OPG **(B)** showing an osteodestructive mass affecting the entire right horizontal mandibular branch infiltrating the lingual and vestibular cortex. **(C, D)** Histologically, the tumour is biphasic and consists of an ameloblastoma-like epithelial proliferation embedded in a hypercellular stroma, which alternates with more hypocellular areas. In the cell-rich component of the stroma, high mitotic activity and nuclear atypia are present **(D)**. **(E)** MRI scan of an ameloblastic fibro-dentinosarcoma documenting an expansile mass in the region of the right mandible, which breaches the cortex and extends into the adjacent structures. **(F, G)** Histologically, the tumour shows a biphasic appearance with an ameloblastoma-like epithelial component with aberrant dentin formation. **(H)** The ectomesenchymal component consists of a hypercellular spindle cell stroma in which cytological atypia and increased mitotic activity can be traced.

Odontogenic sarcomas (ODSs) are a group of mixed malignant odontogenic neoplasms in which only the ectomesenchymal component exhibits morphological features of malignancy. Ameloblastic fibrosarcoma is the most common ODS (38, 39). These neoplasms are extremely rare, with less than 100 cases reported in the literature (39). The peak incidence is in the third decade of life with a slight male predominance, and the most common location is in the mandible (39). Radiologically, most lesions show osteolytic, uni- or multilocular masses with cortical destruction (40) (**Figures 6A, B, E**). Histologically, odontogenic sarcomas show a biphasic pattern with an epithelial and ectomesenchymal component. The epithelial component is, by definition, benign, and often shows large aggregates of odontogenic epithelium with ameloblastic differentiation including nuclear palisading, subnuclear vacuoles and reverse polarity (**Figures 6C, D**). The ectomesenchymal component shows hypercellularity, cytological atypia and increased mitotic activity. Rarely, dysplastic dentine with or without enamel matrix can be seen, in which case the designated nomenclature is ameloblastic fibro-dentinosarcoma and ameloblastic fibro-odntosarcoma, respectively (**Figures 6F–H**). It is believed that more than half of odontogenic sarcomas arise from a pre-existing benign odontogenic neoplasm, such as ameloblastic fibroma. In most cases activating p.V600E mutation in the *BRAF* gene are present in the mesenchymal component (41). In cases negative for *BRAF, NRAS* mutations have been described (42).

Odontogenic carcinosarcoma (OCS) is also a malignant mixed odontogenic tumour. In contract to odontogenic sarcoma, both the epithelial as well as the ectomesenchymal components are malignant. The posterior mandible is the most commonly affected bone (43). OCS are exceedingly rare tumours and the data available are based only on few case reports. As for odontogenic sarcoma, OCS are believed to arise from a pre-existing odontogenic neoplasm, often ameloblastic fibroma (44). Radiologically OCS present as large, destructive, ill-defined masses, often with soft tissue extension (45). As mentioned above, from a histological point of view, both components must show unequivocal features of malignancy: cytological atypia, increased mitotic figures and/or tumour necrosis. The major differential diagnosis of OCS are malignant epithelial odontogenic tumours exhibiting a spindle cell morphology, such as ameloblastic carcinoma. Almost two thirds of patients experiment recurrences, and about 40% metastatic disease (44).

## Discussion/Conclusion

Craniofacial bone sarcomas represent a heterogeneous group of challenging lesions that require specific expertise for proper classification. The morphology remains the backbone for diagnosis, but molecular testing can be useful for certain differential diagnoses, such as *IDH1/IDH2* mutations for distinguishing between chondroblastic OS and chondrosarcoma, as well as *MDM2* amplification and *GNAS* mutations for discriminating between low-grade OS and fibro-osseous mimics. Some entities like MCS or RMS-TFCP2 show a striking predilection for the craniofacial bones, and, interestingly, high-grade OS of jaws are associated with a better prognosis compared to their peripheral counterparts. Chordomas have significant histological variability and the differential diagnosis includes numerous entities, such as low-grade chondrosarcoma or malignant rhabdoid tumour. Experiences with targeted treatment approaches are still limited but molecular testing should be encouraged to shed light on the mechanisms underlying these rare tumours and to identify molecular targets.

## Author Contributions

DB conceived the article. SH, VA, and DB wrote the article. All authors contributed to the article and approved the submitted version.

## Funding

SH, VA, and DB were supported by the Swiss National Science Foundation, the Foundation of the Bone Tumour Reference Centre, the Gertrude von Meissner Stiftung and the Stiftung für krebskranke Kinder, Regio Basiliensis.

## Conflict of Interest

The authors declare that the research was conducted in the absence of any commercial or financial relationships that could be construed as a potential conflict of interest.

## Publisher’s Note

All claims expressed in this article are solely those of the authors and do not necessarily represent those of their affiliated organizations, or those of the publisher, the editors and the reviewers. Any product that may be evaluated in this article, or claim that may be made by its manufacturer, is not guaranteed or endorsed by the publisher.
